# White-box software test generation with Microsoft Pex on open source C# projects: A dataset

**DOI:** 10.1016/j.dib.2020.105962

**Published:** 2020-07-03

**Authors:** Dávid Honfi, Zoltán Micskei

**Affiliations:** a Department of Measurement and Information Systems, Budapest University of Technology and Economics, Budapest, Hungary

**Keywords:** Software testing, Test generation, Symbolic execution, Code coverage, White-box testing

## Abstract

The paper presents a dataset on software tests generated using the Microsoft Pex (IntelliTest) test generator tool for 10 open source projects. The projects were selected randomly from popular GitHub repositories written in C#. The selected projects contain 7187 methods from which Pex was able to generate tests for 2596 methods totaling 38,618 lines of code. Data collection was performed on a cloud virtual machine. The dataset presents metrics about the attributes of the selected projects (e.g., cyclomatic complexity or number of external method calls) and the test generation (e.g., statement and branch coverage, number of warnings). This data is compared to an automated isolation technique in the paper Automated Isolation for White-box Test Generation [1]. To the best of our knowledge, this is the largest public dataset about the test generation performance of Microsoft Pex on open source projects. The dataset highlights current practical challenges and can be used as a baseline for new test generation techniques.

Specifications table

**Table:** 

Subject	Computer Science / Software
Specific subject area	Software testing
Type of data	Table
	Plot
How data were acquired	Microsoft Pex was executed on the selected projects repeatedly one method per time. Data were collected using scripts parsing the output reports and generated tests.
Data format	Raw
	Analyzed
Parameters for data collection	Test generation was executed on 10 different open source projects selected from GitHub 1) using the default parameters of Pex and then 2) with the automated isolation technique recommended in [1].
Description of data collection	Test generation was executed on a cloud virtual machine running Windows 10 and having 3.5 GBs of memory along with a dedicated CPU running at 2.4 GHz. The version of Pex was 15.0.27005.2 (shipped with Visual Studio 2017 version 15.8.1). Test generation was repeated three times, and the median values are reported.
Data source location	Data was collected at the Budapest University of Technology and Economics, Budapest, Hungary.
Data accessibility	Repository name: Zenodo
	Data identification number: 10.5281/zenodo.3457479
	Direct URL to data: http://doi.org/10.5281/zenodo.3457479
Related research article	David Honfi, Zoltan Micskei. Automated Isolation for White-box Test Generation. Information and Software Technology. Volume 125, September 2020, 106,319. DOI: 10.1016/j.infsof.2020.106319

## Value of the data

• Large public dataset on the performance of Microsoft Pex test generator and attributes of .NET/C# open source projects
• Can be used by researchers working on test generation techniques or by practitioners considering automated test generation
• Data can be used as a baseline for Microsoft Pex, and can show unsolved challenges experienced on real-world software
• Contains diverse projects highlighting the frequency of different types of issues during test generation

## 1 Data description

### 1.1 Overview of the selected projects

The projects were downloaded from their respective public GitHub repository in August 2019. Some of the projects were not the latest versions, or they had to be modified to comply with Microsoft Pex's capabilities [2], [4]. The dataset contains information about 10 projects with 698 classes and 2596 methods (Table 1). Methods retained in the dataset must satisfy two criteria: i) Pex was able to start test generation (e.g., they are public, they are in a non-abstract class, etc.) and ii) our tool presented in [1] did not throw any error. Note that a large number of methods were filtered from the initial 7187 because of the current limitations of Pex.

**Table 1:** The projects from GitHub used in the dataset.

Project	Classes	Methods
abot-master^1^	21	57
akka.net^2^	220	909
GraphEngine^3^	54	233
Humanizer^4^	57	107
ImageProcessor-develop^5^	81	182
LiteDB^6^	50	263
nodatime-2^7^	42	199
Polly-master^8^	16	47
Simple.Data-master^9^	71	363
Topshelf^10^	86	236
*SUM*	*698*	*2596*

1 https://github.com/sjdirect/abot.

2 https://github.com/akkadotnet/akka.net.

3 https://github.com/microsoft/GraphEngine.

4 https://github.com/Humanizr/Humanizer.

5 https://github.com/JimBobSquarePants/ImageProcessor.

6 https://github.com/mbdavid/LiteDB.

7 https://github.com/nodatime/nodatime.

8 https://github.com/App-vNext/Polly.

9 https://github.com/markrendle/Simple.Data.

10 https://github.com/Topshelf/Topshelf.

**Fig. 1:**
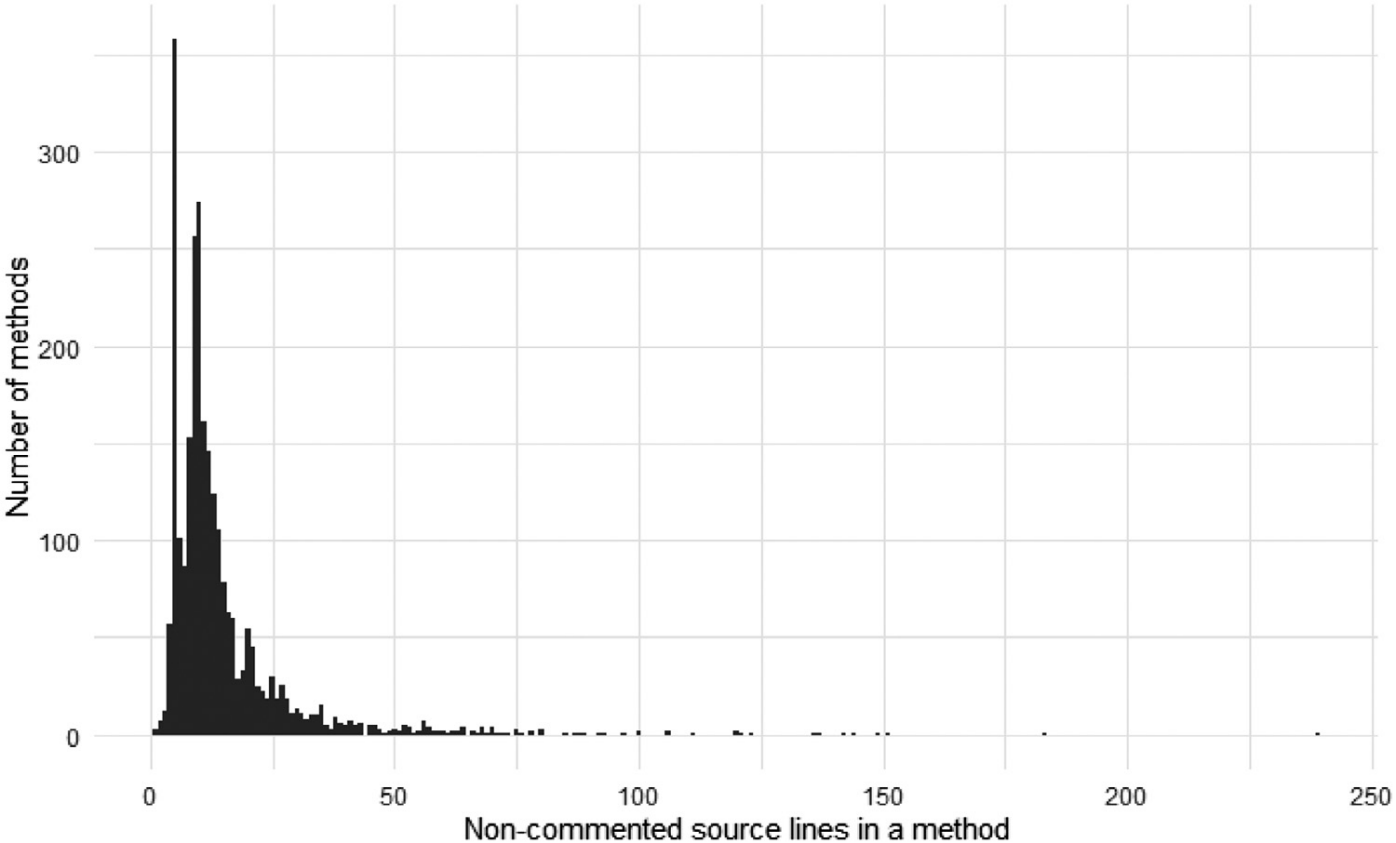
The distribution of the methods’ length measured with lines of code.

**Fig. 2:**
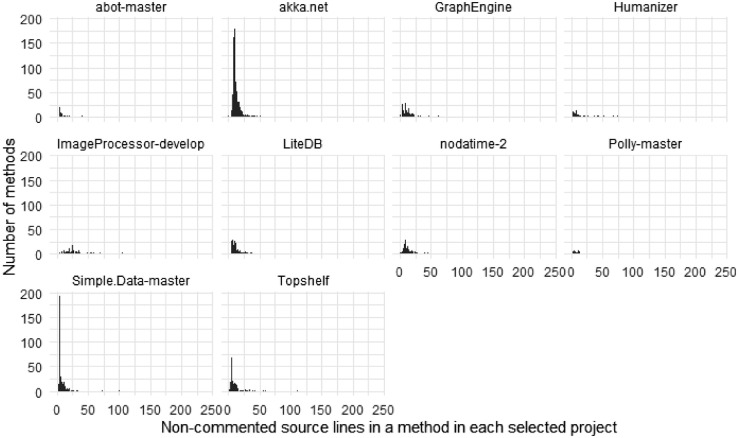
The distribution of methods’ lengths per project measured in lines of code.

Most of the methods are rather short; however, there are some outliers. Fig. 1 shows the distribution of the non-commented source lines of code (LoC) found in the methods. Fig. 2 presents the same distribution separated by projects. Note the tail and the outliers: several methods were having more than 100 LoC.

**Fig. 3:**
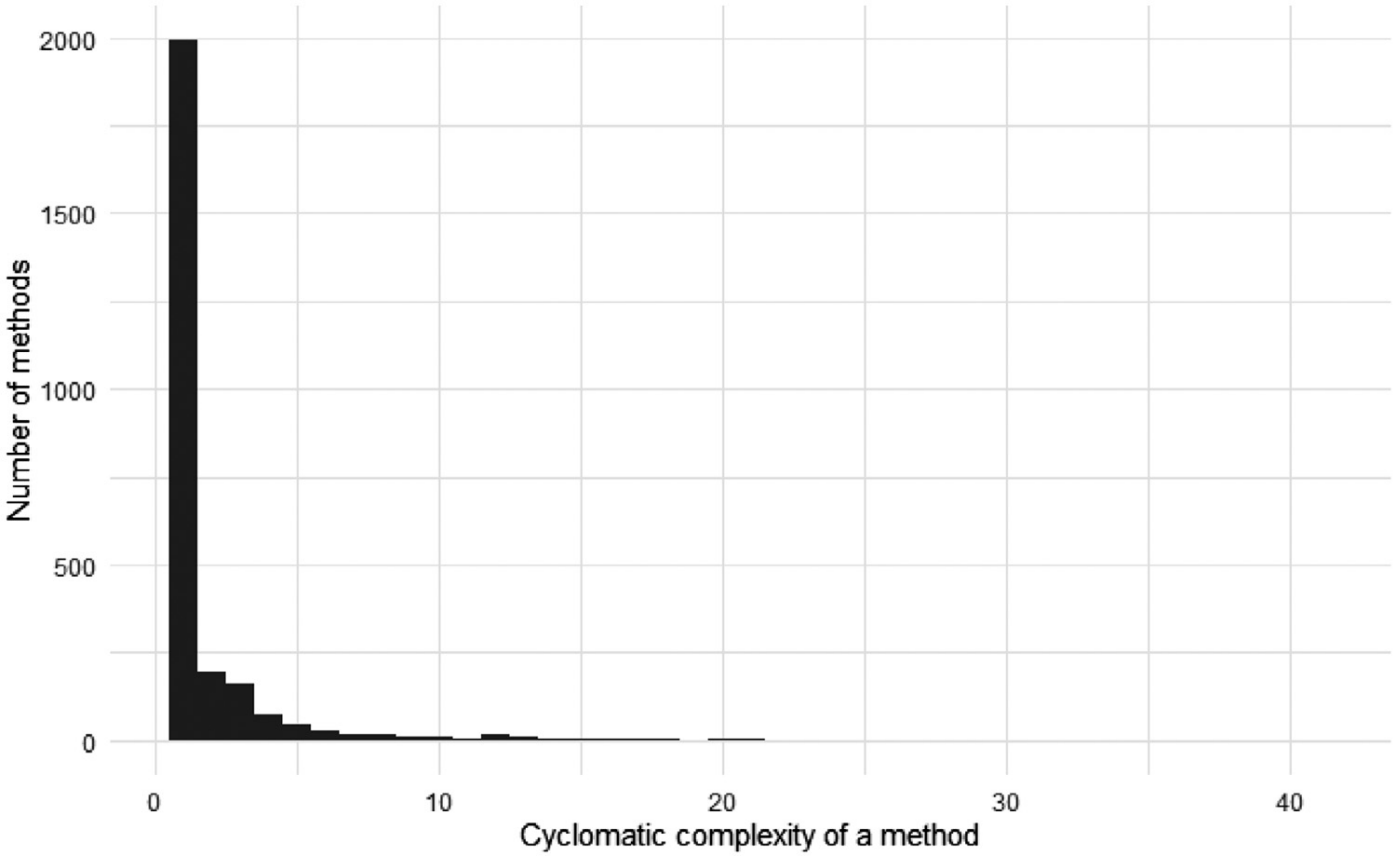
The distribution of the methods’ complexity measured with cyclomatic complexity.

**Fig. 4:**
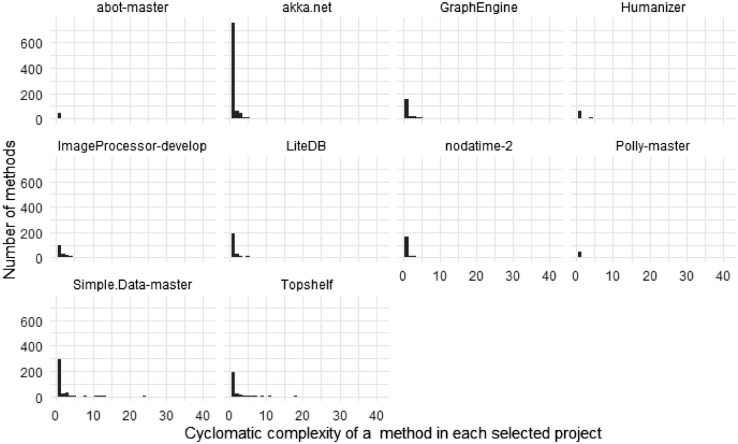
The distribution of methods’ complexity per project measured with cyclomatic complexity.

Fig. 3, Fig. 4 depict the distribution of the summarized and the per-project complexity, respectively. The complexity of a method is measured by its cyclomatic complexity, which was computed using Roslyn [3].

**Fig. 5:**
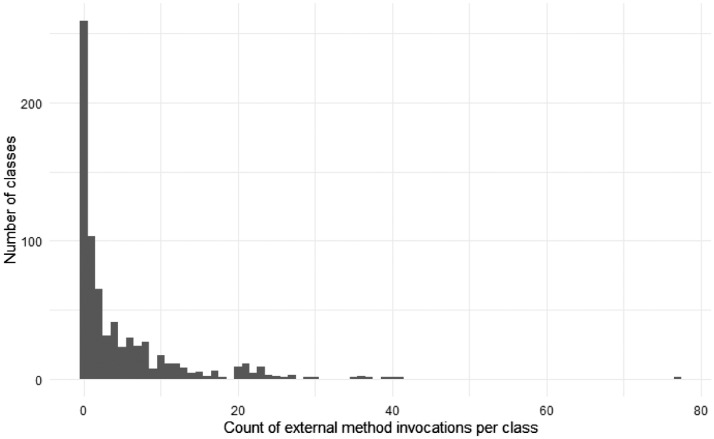
The number of external method invocations in each class under test.

**Fig. 6:**
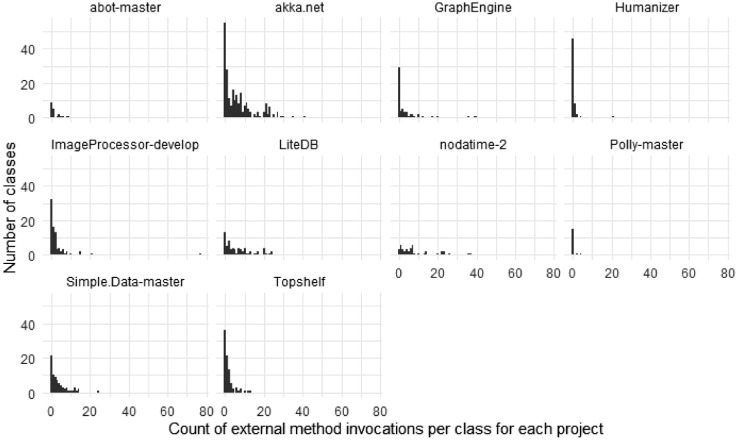
The number of external method invocations in each class under test per project.

**Fig. 7:**
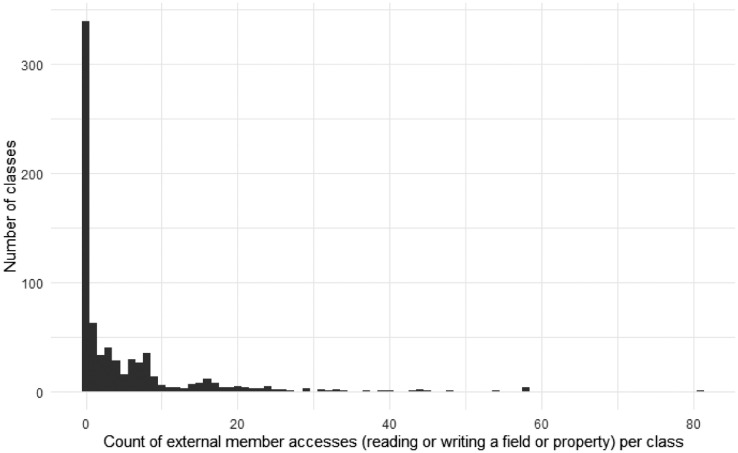
The number of external member accesses in each unit under test.

**Fig. 8:**
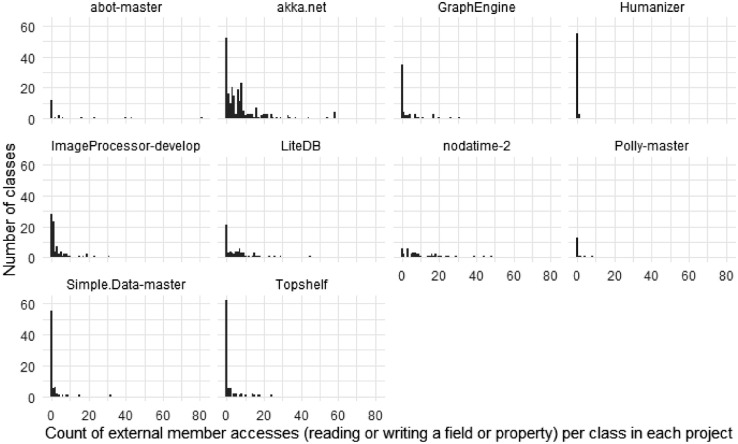
The number of external member accesses in each unit under test per project.

Fig. 5, Fig. 6, and Fig. 7, Fig. 8 give insights on how many dependencies the classes in the selected projects have in terms of individual method invocations and member accesses (reading or writing a field or property), respectively. These dependencies are counted using abstract syntax tree traversal by comparing the invocations and member accesses to the fully-qualified definition of the class under test. Note that some calls to the system libraries are not counted as external as those are necessary to have valid functionalities (e.g., date handling is counted as external, while string operations are not). Note that some classes had more than 70 external method invocations or member accesses.

### 1.2 Overview of the generated tests and the coverage reached

**Fig. 9:**
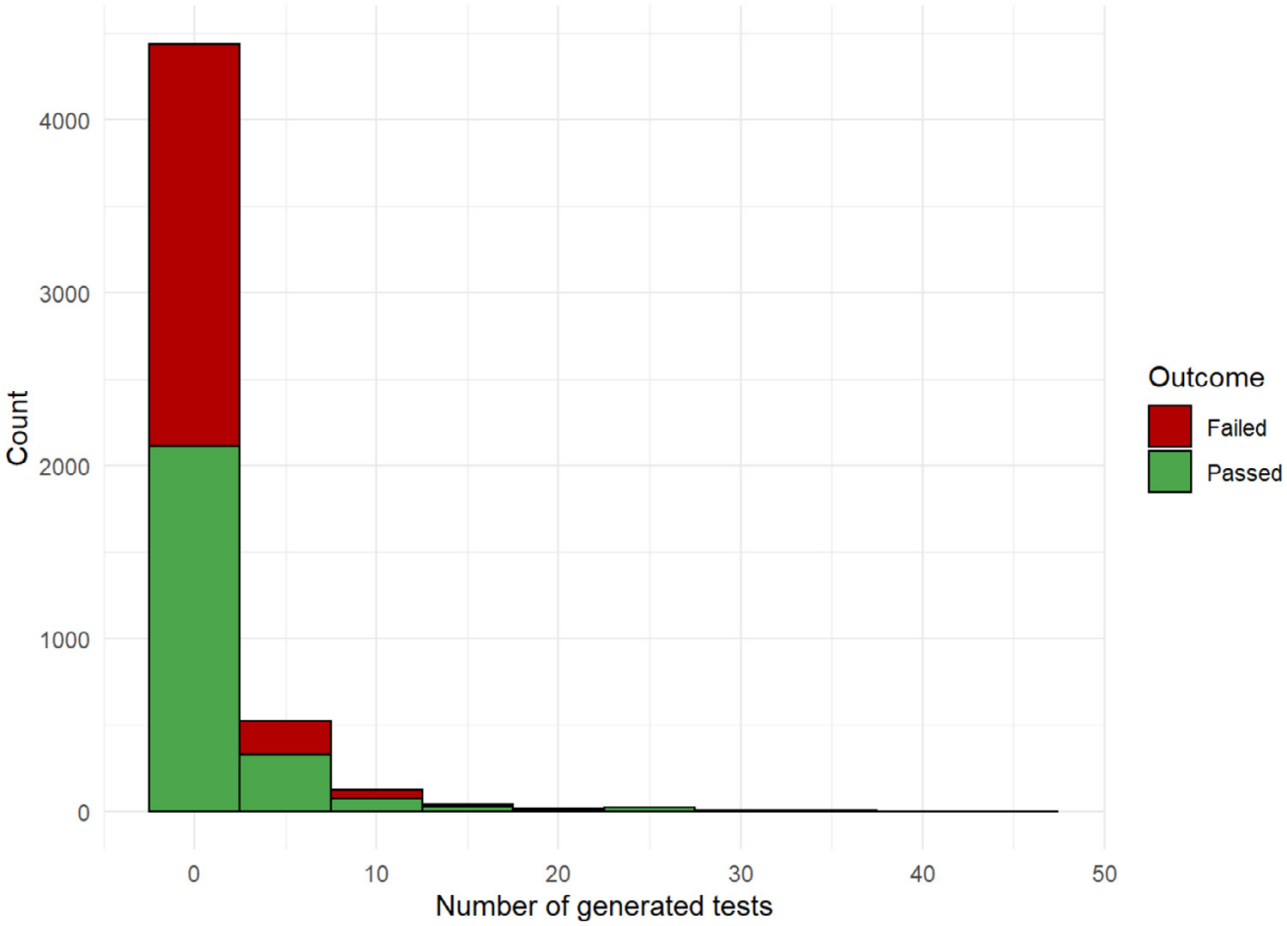
The distribution of the number of generated tests for each method.

The number of generated tests by Pex fall into three categories (see [1]): no tests were generated (864), a single test case was generated (793), multiple test cases were generated (939). The number of generated tests is an initial indicator of the success of test generation. Fig. 9 depicts the distribution of the number of passed and failed generated tests per method. A generated test fails, if it throws an unexpected exception (i.e., not explicitly thrown by the class under test).

**Fig. 10:**
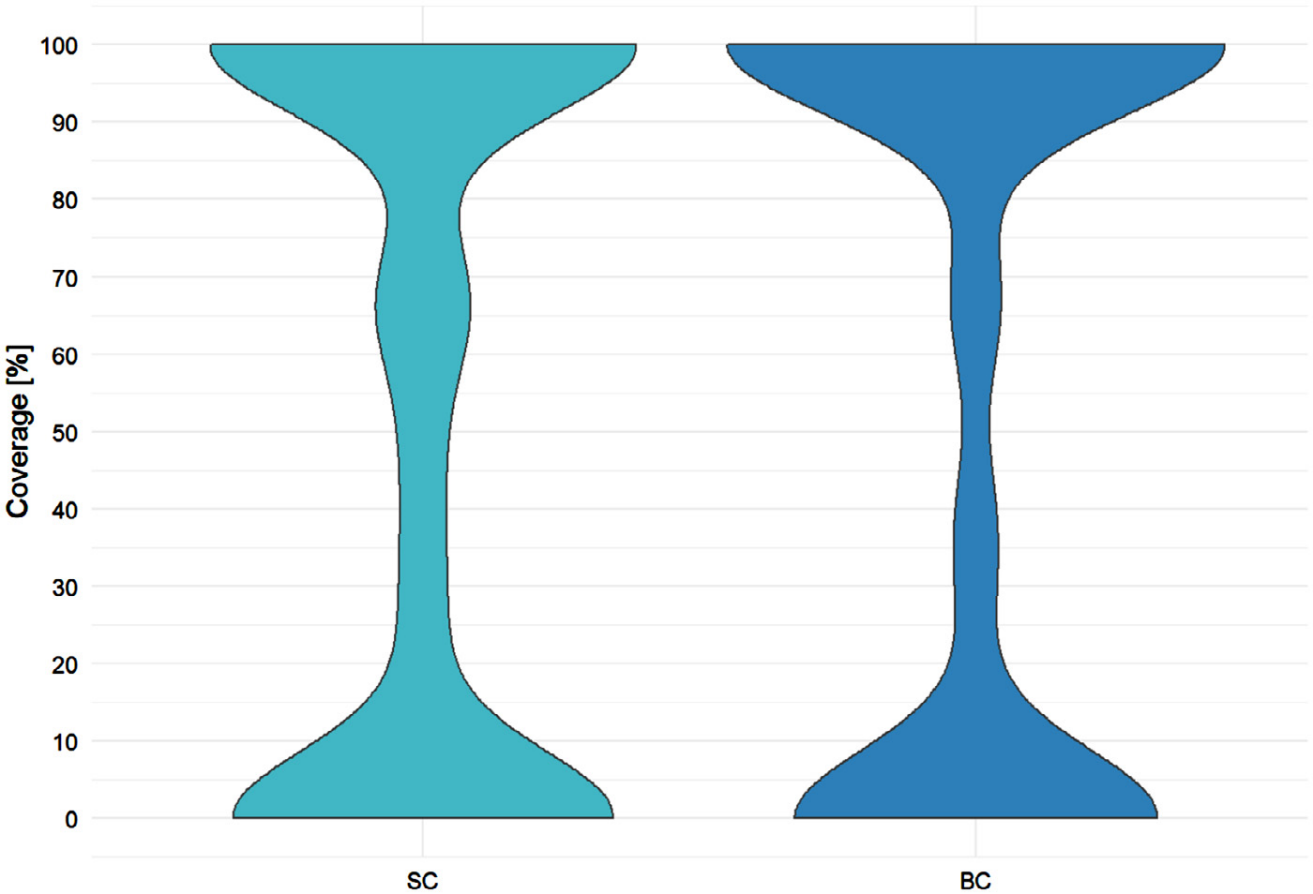
Violin plots for statement (SC) and branch coverage (BC) reached by generated tests of Pex.

Fig. 10 shows the violin plots for statement (SC) and branch coverage (BC) percentage reached by generated tests in total. Note the thickenings at the range ends for both statistics showing that for most of the methods either 0% or 100% coverage is reached.

**Fig. 11:**
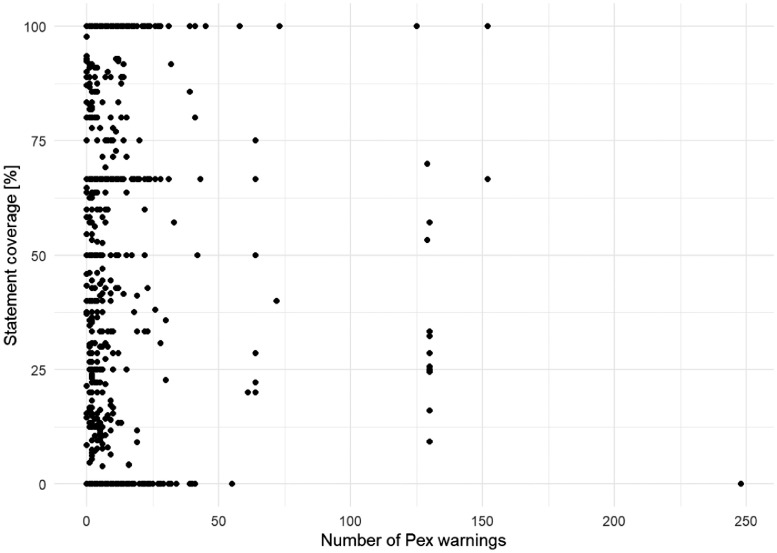
The scatterplot of the number of warnings reported by Pex versus statement coverage.

**Fig. 12:**
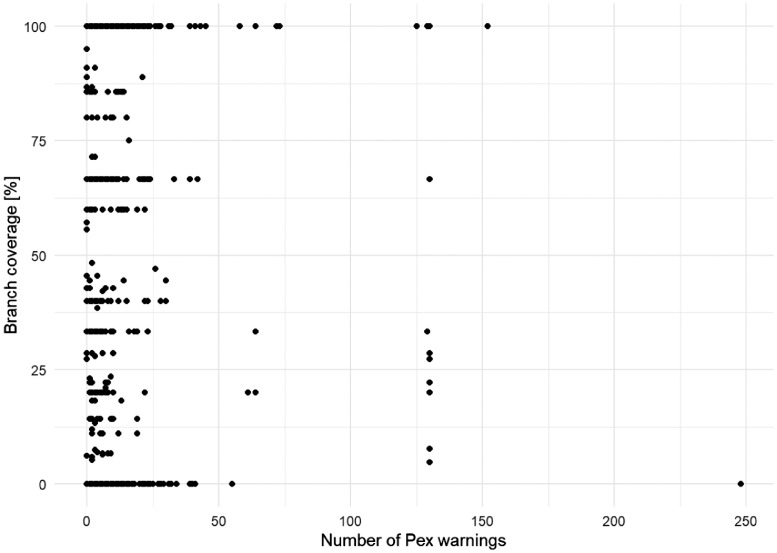
The scatterplot of the number of warnings reported by Pex versus branch coverage.

Fig. 11, Fig. 12 present the relationship between the two kinds of coverages measured (statement and branch coverage) and the number of warnings reported by Microsoft Pex throughout its test generation process.

**Fig. 13:**
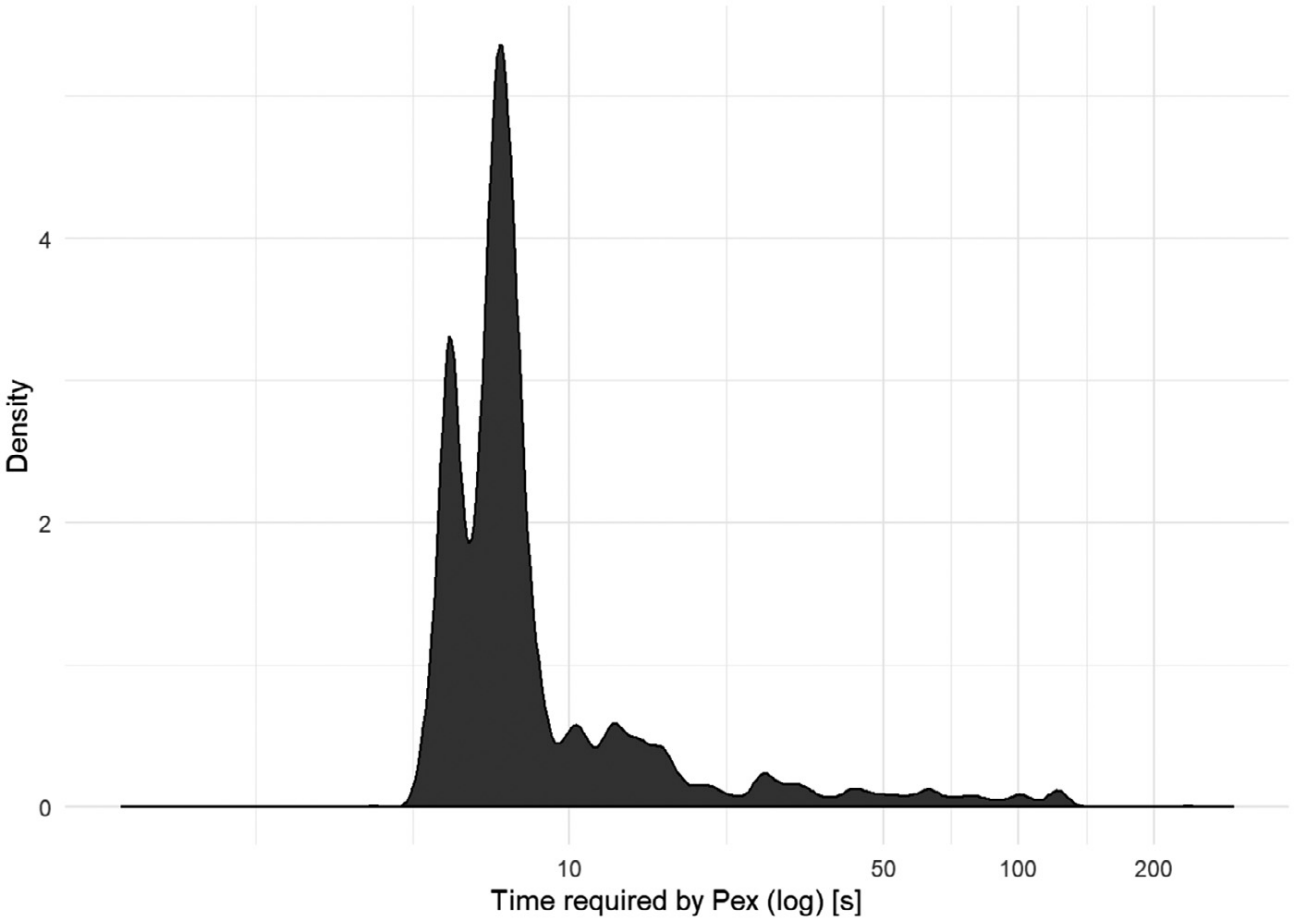
The density plot of the time required by Pex for the whole test generation process.

Fig. 13 shows the density of the time consumption of Pex. In most of the cases, Pex required less than 10 s to generate the test cases.

## 2 Experimental design, materials, and methods

We selected the experimental design variables by separating them into dependent (controlled) and independent (observed) ones. There were 7 independent variables: statement coverage, branch coverage, number of generated tests, number of warnings reported by Pex, number of external method invocations, number of external member accesses, the time required for test generation. The study design controlled only the method under test.

The 10 projects that contain the executed methods were randomly selected from GitHub using the predefined criteria, which specified constraints on the number of stars given for the repository (> = 1000), on the non-existence of a relation to user interfaces, mobile applications, or graphical components. The methods from those projects (repositories) were automatically extracted, followed by another filtering based on criteria defined for methods (or their containing classes): no nested classes, no abstract classes, no generic classes, no abstract methods, no extension methods, and finally no non-public methods.

During the experiment we performed each measurement 3 times to ensure the validity of data collection from which the median values were extracted automatically. The experiment was executed on a virtual machine having 3.5 GBs of memory and a dedicated CPU running at 2.4 GHz. The data was analyzed with R 3.4.3 [5], including the extraction of descriptive statistics and the exploratory data analysis.

## Declaration of Competing Interest

The authors declare that they have no known competing financial interests or personal relationships which have, or could be perceived to have, influenced the work reported in this article.
